# Epidemiological insights into severe hepatitis in pediatric inpatients

**DOI:** 10.1016/j.isci.2024.111420

**Published:** 2024-11-19

**Authors:** Fuping Guo, Xudong Ma, Qijun Shan, Yuelun Zhang, Sifa Gao, Jieqin Chen, Yujie Chen, Dawei Liu, Taisheng Li, Feng Zhang, Xiang Zhou, the China Critical Care Clinical Trials Group (CCCCTG) and China National Critical Care Quality Control Center Group

**Affiliations:** 1 Department of Infectious Diseases, Peking Union Medical College Hospital, Chinese Academy of Medical Sciences, Beijing 100730, China; 2 Department of Medical Administration, National Health Commission of the People’s Republic of China, Beijing 100044, China; 3 Department of Information Center, Peking Union Medical College Hospital, Chinese Academy of Medical Sciences, Beijing 100730, China; 4 Central Research Laboratory, Peking Union Medical College Hospital, Chinese Academy of Medical Sciences, Beijing 100730, China; 5 Department of Critical Care Medicine, Peking Union Medical College Hospital, Chinese Academy of Medical Sciences, Beijing 100730, China

**Keywords:** Health sciences, medicine, hepatology

## Abstract

There is limited statistical data on severe hepatitis among hospitalized children in China at a national level. This nationwide study analyzed data from 34,410 children hospitalized for severe hepatitis in mainland China from 2016 to 2022. The overall in-hospital mortality rate was 6.1%. Viral hepatitis accounted for 14.9% of cases, with hepatitis B being the most prevalent. Indeterminate etiology, present in 79.0% of cases, was an independent risk factor for mortality. Findings emphasize the need for enhanced diagnostic methods and targeted interventions for children with severe hepatitis of unknown cause and suggest focused hepatitis B monitoring in older children to improve outcomes. Establishing a dedicated surveillance system is crucial for improving the prognosis of children at high risk for in-hospital mortality due to severe hepatitis.

## Highlights

• First nationwide epidemiological study on pediatric severe hepatitis in mainland China
• Indeterminate causes comprised 79% of cases, significantly raising in-hospital mortality risk
• Hepatitis B prevalence rose with age, underscoring the need for better monitoring
• Enhanced diagnostics like liver biopsy and NGS are needed to reduce indeterminate cases

## Graphical abstract

**Figure:**
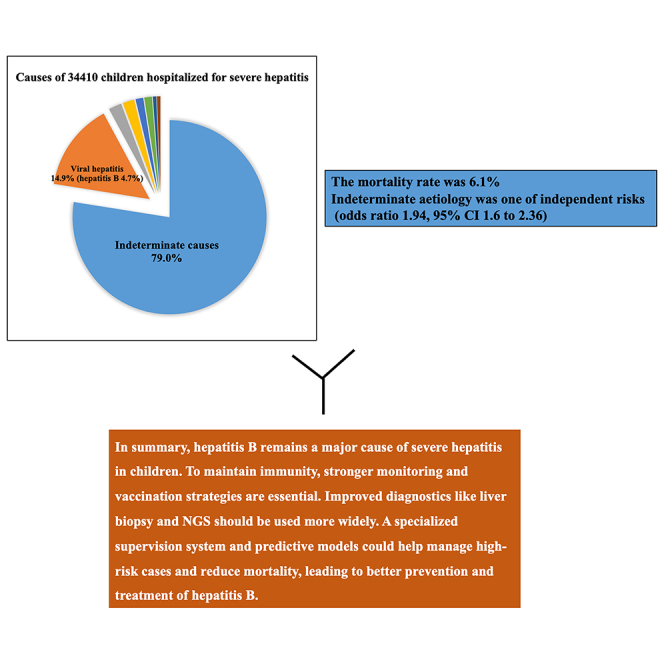

## Introduction

Following the United Kingdom report of 10 pediatric cases of acute severe hepatitis of unknown etiology,^1^ this condition has gradually received widespread attention. Since then, many cases have been found in many countries. As of 8 July 2022, 35 countries have reported 1,010 cases of probable severe acute hepatitis of unknown etiology among children according to a report from the World Health Organization (WHO), including 46 (4.6%) cases requiring liver transplants and 22 (2.2%) deaths.^2^ This outbreak of acute severe hepatitis in children was characterized by unknown etiology and high mortality. The possible causes were adenovirus 41F, severe acute respiratory syndrome coronavirus 2 (SARS-CoV-2) superantigens,^3^ and autoimmune-like hepatitis after mRNA coronavirus disease 2019 (COVID-19) vaccination.^4^ However, few cases have been reported in Asia, especially in China.

Despite having one of the world’s largest pediatric populations, there is limited statistical data on severe hepatitis among hospitalized children in China at a national level. Previous studies have primarily focused on adult populations or were limited to specific regions, leaving a considerable data gap in understanding the burden of severe pediatric hepatitis at a national level. This study addresses this critical gap by providing the first nationwide epidemiological analysis of children hospitalized for severe hepatitis in China.

To address the potential risk of outbreaks involving acute severe hepatitis of unknown etiology in Chinese children, it is crucial to understand the current landscape of pediatric severe hepatitis nationwide. This study aims to analyze the epidemiological characteristics and mortality-related risk factors for children hospitalized with severe hepatitis across mainland China using a large-scale, nationwide real-world dataset. By leveraging the data from the children hepatopathy in China study (CHPC study), this research provides early warning information and informs treatment strategies for future outbreaks among children hospitalized for severe hepatitis.

### Materials and methods

#### Patients and study design

Detailed data on severe hepatitis hospitalizations were collected from the database of the Hospital Quality Monitoring System (HQMS) of the National Health Commission of the People’s Republic of China (https://www.hqms.org.cn/login.jsp). In 2016, this database included patient medical records from 2,405 nationwide secondary hospitals and above. An approximately five-year interval from January 2016 to April 2022 was selected for full analysis according to International Classification of Diseases, Tenth Revision (ICD-10) diagnosis codes. The inclusion criteria for patient enrollment were the following: (1) aged younger than 18 years; (2) hospitalized for severe hepatitis defined by ICD-10 diagnosis codes (B15–19, K72) (Table S1).

We extracted all demographic data as follows: age, sex, ethnicity, place of residence, hospital type, diagnosis at discharge, date of hospitalization, discharge date, and hospitalization costs. We then identified the cause of hospitalization for severe hepatitis as follows: hepatitis A, hepatitis B, hepatitis C, hepatitis E, cytomegalovirus (CMV) infection, Epstein-Barr (EBV) infection, adenovirus infection, human immunodeficiency virus (HIV) infection, fatty liver disease, alcoholic hepatitis, drug-induced liver injury, autoimmune hepatitis, congenital hepatitis, Wilson disease, liver tumor, biliary cirrhosis and indeterminate causes. We further included the following clinical data: hepatic encephalopathy, gastrointestinal bleeding, ascites, coagulopathy, liver failure, acute kidney failure, acute respiratory failure, brain death, ICU length of stay, ventilation time, hemodialysis, use of artificial liver, liver biopsy, liver transplantation, and death. ICD-10 encodings for causes and complications of severe hepatitis were shown (Table S2).

We grouped patients according to age as follows: less than 5, 5–9, 10–14, and 15–17 years. The 31 provinces/municipalities/autonomous regions of mainland China were included in this study (data from Hong Kong, Taiwan, and Macao were not included).

China is divided into seven geographic regions: Central China, North China, East China, South China, Northwest China, Northeast China, and Southwest China. Three economic zones were also analyzed, including southeastern coastal areas, central inland areas, and western remote areas. According to the level of gross domestic product (GDP), China was divided into high-, middle-, and low-GDP regions (Table S3). The costs are expressed in USD, and the exchange rate between RMB and USD was based on the standard exchange rate on January 1, 2022 (1 USD to 6.7467 RMB).

#### Statistical analysis

Descriptive data were tabulated and reported using simple means, standard deviations, medians, interquartile ranges, and frequencies. Analysis of variance was used for parametric continuous variables, the Kruskal-Wallis rank-sum test was used for nonparametric continuous variables, and the chi-squared test was used for categorical variables.

The incidence rate of severe hepatitis was modeled using the negative binomial model in which the patient number by province and the categorical year were included as dependent and independent variables, respectively. The rate ratios (RRs) with corresponding 95% confidence intervals (CIs) were estimated from the negative binomial model. Because the data in 2022 were from January to April, we analyzed the data from January to April of each year from 2016 to 2022.

Risk factors for mortality among children hospitalized for severe hepatitis were analyzed by logistic regression in multivariable models. The dichotomous outcomes were discharged alive versus death. Potentially relevant risk factors were selected using stepwise regression based on the Akaike information criterion (AIC). The results of the logistic regression were reported by odds ratios (ORs) with 95% CIs.

Pearson or Spearman correlation analyses were performed to examine the relationships between the length of hospital stay, overall cost and death associated with causes and complications of children with severe hepatitis.

All tests performed were two-tailed, with *p* < 0.05 considered to be statistically significant. Statistical calculations were performed using the R program (version 4.2.1, R Core Team, 2022. R Core Team, 2020. R: A language and environment for statistical computing. R Foundation for Statistical Computing, Vienna, Austria. https://www.R-project.org/).

## Results

**Figure 1:**
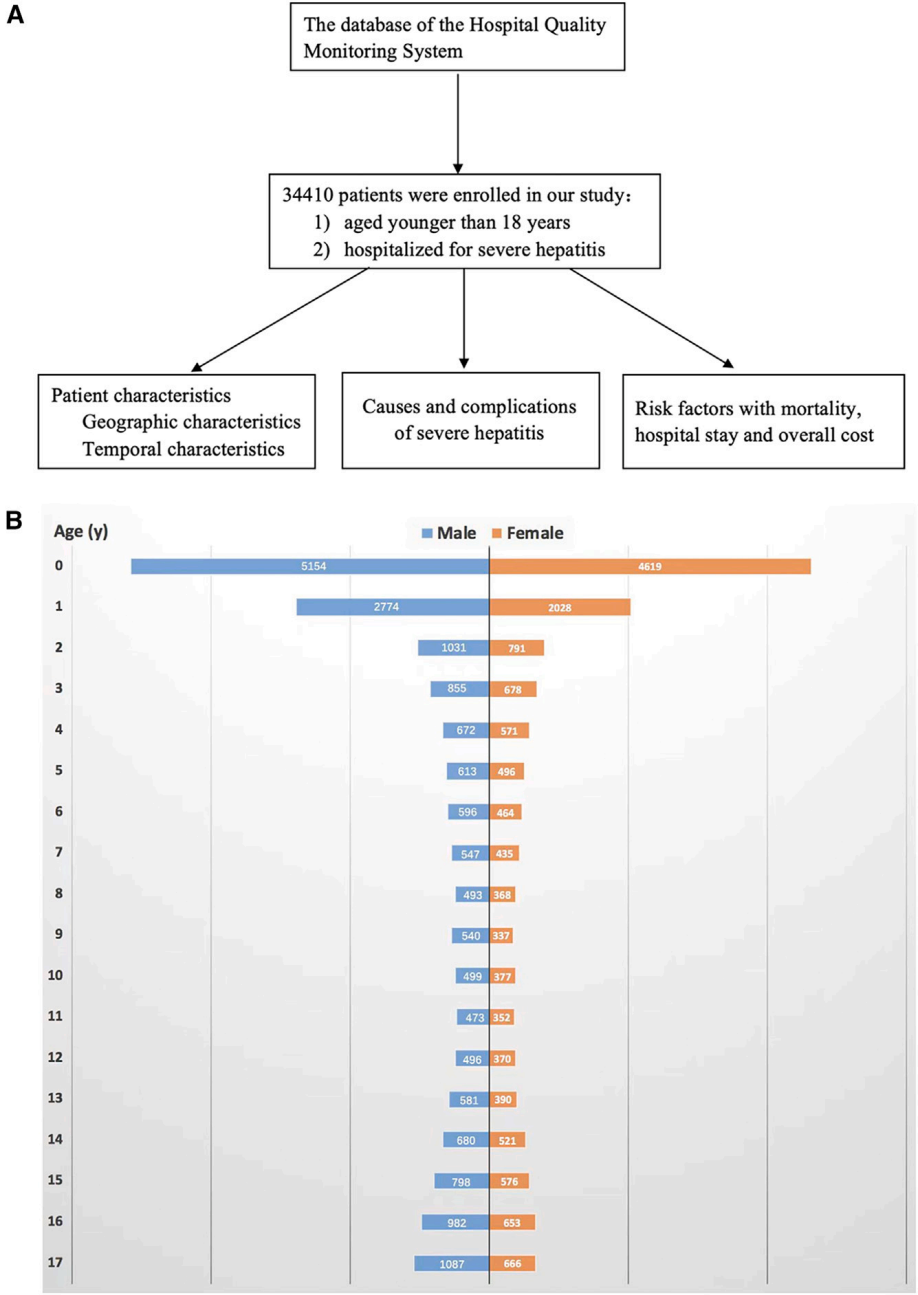
Study flowchart and demographic distribution of children hospitalized for severe hepatitis (*n* = 34,410) (A) Flowchart of study. (B) Age and sex distribution among the 34410 children hospitalized for severe hepatitis.

A total of 34410 children hospitalized for severe hepatitis with data included in the HQMS database were enrolled in our study beginning in 2016 (Figure 1A). According to the age groups (<5 years, 5–9 years, 10–14 years, and ≥15–17 years), the numbers of cases were 19816 (57.6%), 4986 (14.5%), 4803 (14.0%), and 4805 (14.0%), respectively. Patients were from 2,405 hospitals, including 1,045 secondary hospitals and 1,360 tertiary hospitals.

### Patient characteristics

The demographic characteristics of the children hospitalized for severe hepatitis are shown in Table 1. The average age of the children hospitalized for severe hepatitis was 5.5 ± 6.0 years, and 18871 (54.8%) patients were male. Children aged <5 years accounted for 57.6%, and nearly one-third (28.4%) of children were aged <1 year (Figure 1B). Most patients were of Han ethnicity (84.1%). Most patients (87.8%) were admitted to tertiary hospitals. The median length of hospital stay was 8 days (5, 14). Nearly two-thirds of children were hospitalized for less than 10 days, and older children had a significantly longer hospital stay (*p* < 0.001). The median hospital cost was 1183 (563, 3890) USD. More than half of the children had a hospital cost lower than 1482 USD, and older children had a significantly higher cost (*p* < 0.001).

**Table 1:** Demographic characteristics of children hospitalized for severe hepatitis nationwide

	Total	<5 years	5-9 years	10-14 years	≥15–17 years	P
N	34410	19816	4986	4803	4805	–
Age (years)	5.5 ± 6.0	0.9 ± 1.2	6.9 ± 1.4	12.2 ± 1.5	16.1 ± 0.8	<0.001
Male	18871 (56.2)	10486 (54.7)	2789 (57.0)	2729 (57.6)	2867 (60.2)	<0.001
Han ethnicity	28904 (84.1)	16762 (84.7)	4096 (82.3)	4061 (84.8)	3985 (83.2)	<0.001
Geographical area	–	–	–	–	–	<0.001
Northeast	790 (2.3)	328 (1.7)	120 (2.4)	163 (3.4)	179 (3.7)	–
North China	2513 (7.3)	1188 (6.0)	419 (8.4)	504 (10.5)	402 (8.4)	–
East China	8718 (25.3)	5317 (26.8)	1229 (24.6)	1156 (24.1)	1016 (21.1)	–
South China	5494 (16.0)	3481 (17.6)	730 (14.6)	617 (12.8)	666 (13.9)	–
Central China	4378 (12.7)	2516 (12.7)	591 (11.9)	623 (13.0)	648 (13.5)	–
Northwest	4827 (14.0)	2966 (15.0)	728 (14.6)	584 (12.2)	549 (11.4)	–
Southwest	7690 (22.3)	4020 (20.3)	1169 (23.4)	1156 (24.1)	1345 (28.1)	–
Economic zones of China	–	–	–	–	–	<0.001
Southeast coastal area	14987 (43.6)	9001 (45.4)	2123 (42.6)	1990 (41.4)	1873 (39.0)	–
Central island area	8861 (25.8)	4954 (25.0)	1247 (25.0)	1353 (28.2)	1307 (27.2)	–
Western remote area	10562 (30.7)	5861 (29.6)	1616 (32.4)	1460 (30.4)	1625 (33.8)	–
Different GDP levels	–	–	–	–	–	<0.001
High	16468 (47.9)	9845 (49.7)	2239 (44.9)	2181 (45.4)	2203 (45.8)	–
Middle	9807 (28.5)	5213 (26.3)	1441 (28.9)	1619 (33.7)	1534 (31.9)	–
Low	8135 (23.6)	4758 (24.0)	1306 (26.2)	1003 (20.9)	1068 (22.2)	–
Type of hospital	–	–	–	–	–	<0.001
Secondary	4201 (12.2)	2231 (11.3)	565 (11.3)	643 (13.4)	762 (15.9)	–
Tertiary	30209 (87.8)	17585 (88.7)	4421 (88.7)	4160 (86.6)	4043 (84.1)	–
Infectious disease hospital	1051(3.1)	219(1.1)	172(3.4)	323(6.7)	337(7.0)	<0.001
Children’s hospital	5343(15.5)	3762(19.0)	939(18.8)	583(12.1)	59(1.2)	<0.001
Hospital stay (days)	8 (5, 14)	7 (4, 12)	9 (4, 16)	10 (5, 18)	10 (5, 18)	<0.001
1–10	21390 (62.2)	13584 (68.6)	2837 (56.9)	2527 (52.6)	2442 (50.8)	–
11–20	7842 (22.8)	3945 (19.9)	1257 (25.2)	1274 (26.5)	1366 (28.4)	–
21–30	2858 (8.3)	1202 (6.1)	518 (10.4)	578 (12.0)	560 (11.7)	–
>30	2319 (6.7)	1085 (5.5)	373 (7.5)	424 (8.8)	437 (9.1)	–
Overall cost (USD)	1183 (563, 3890)	973 (518, 2965)	1313 (551, 4491)	1782 (701, 5587)	1776 (775 5334)	<0.001
<1482	19309 (56.1)	12314 (62.2)	2643 (53.0)	2193 (45.7)	2159 (44.9)	–
1482-2964	4866 (14.1)	2542 (12.8)	701 (14.1)	766 (16.0)	857 (17.8)	–
2964-4447	2400 (7.0)	1180 (6.0)	389 (7.8)	412 (8.6)	419 (8.7)	–
≥4447	7824 (22.7)	3772 (19.0)	1252 (25.1)	1430 (29.8)	1370 (28.5)	–

### Geographic characteristics

More than 2000 children hospitalized for severe hepatitis were in Guangdong, Guizhou, Xinjiang, Sichuan, Anhui and Hunan provinces, whereas there were fewer than 200 cases in Tibet, Ningxia, Inner Mongolia, Hainan, Jilin, and Heilongjiang. And hospital stay longer than ten days were in Qinghai, Beijing, Jiangxi, Guangxi, and Anhui (Figure S1).

In terms of geographical area, the region with the largest number of children hospitalized for severe hepatitis was East China (25.3%). Northeast and North China accounted for 9.6% of the total cases (Table 1). Regarding economic zones, the southeast coastal areas had almost half of the patients (43.6%), followed by the western remote areas and finally the central island area (Table 1).

### Temporal characteristics

**Figure 2:**
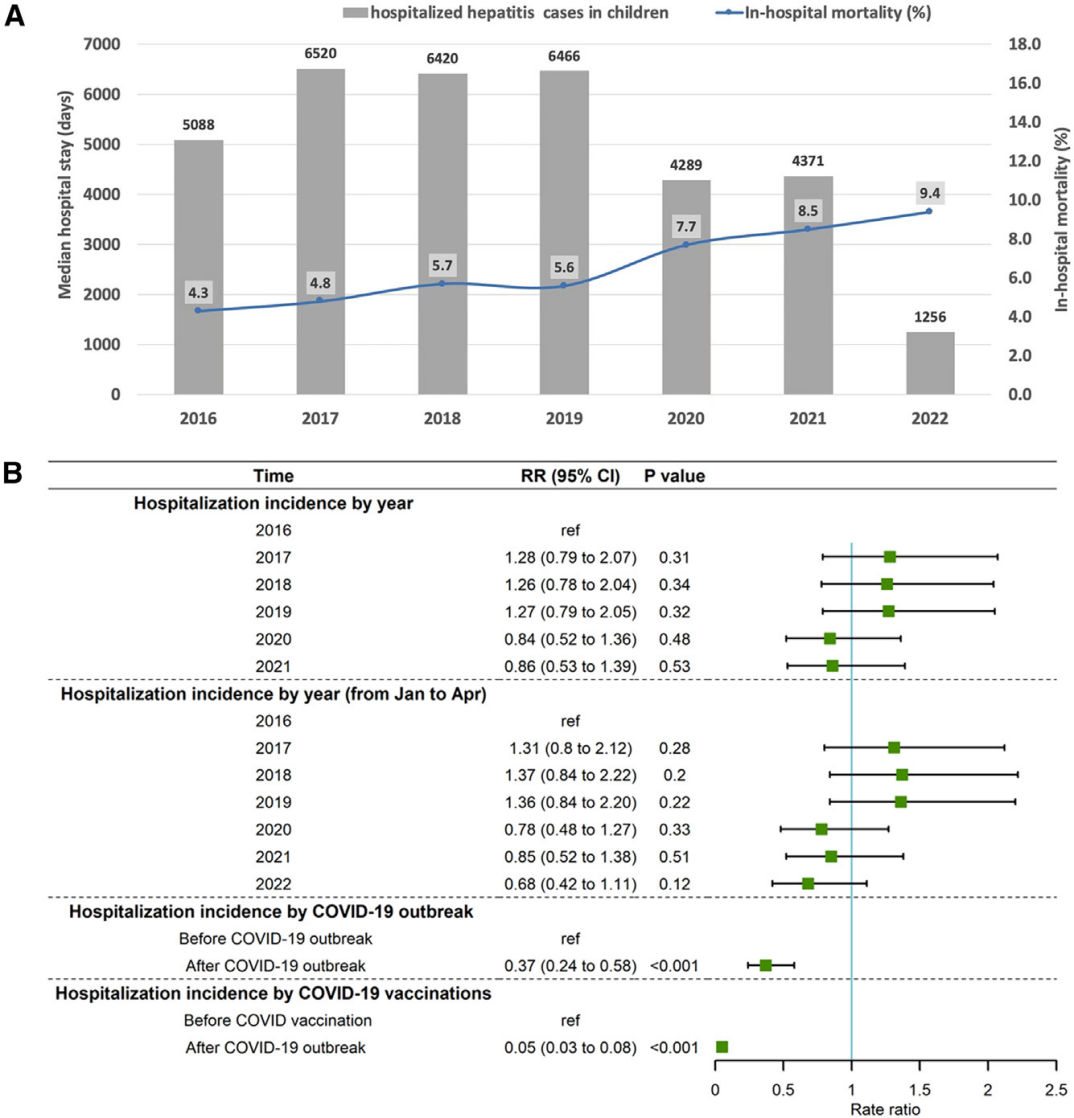
In-hospital mortality and temporal trends of children hospitalized for severe hepatitis in China (2016-2022) (A) In-hospital mortality rate and hospitalized cases of children with severe hepatitis from 2016 to 2022. (B) Temporal characteristics of children hospitalized for severe hepatitis in China; Annual data are from 2016 through 2022, and data in 2022 are from January through April.

The number of hospitalized cases of children with severe hepatitis decreased after the COVID-19 epidemic (Figure 2A). The incidence of hospitalization for severe hepatitis among children did not significantly change annually from 2016 to 2021 (Figure 2B). However, the RRs in 2020 and 2021 showed a potential downward trend. Similar results were demonstrated from January to April annually from 2016 to 2022.

The incidence of children hospitalized for severe hepatitis decreased significantly after the COVID-19 outbreak (RR 0.37, 95% CI 0.24 to 0.58, *p* < 0.001) (Figure 2B). A similar result was found after COVID-19 vaccinations began (RR 0.05, 95% CI 0.03 to 0.08, *p* < 0.001).

### Causes of hospitalization for severe hepatitis among children

**Figure 3:**
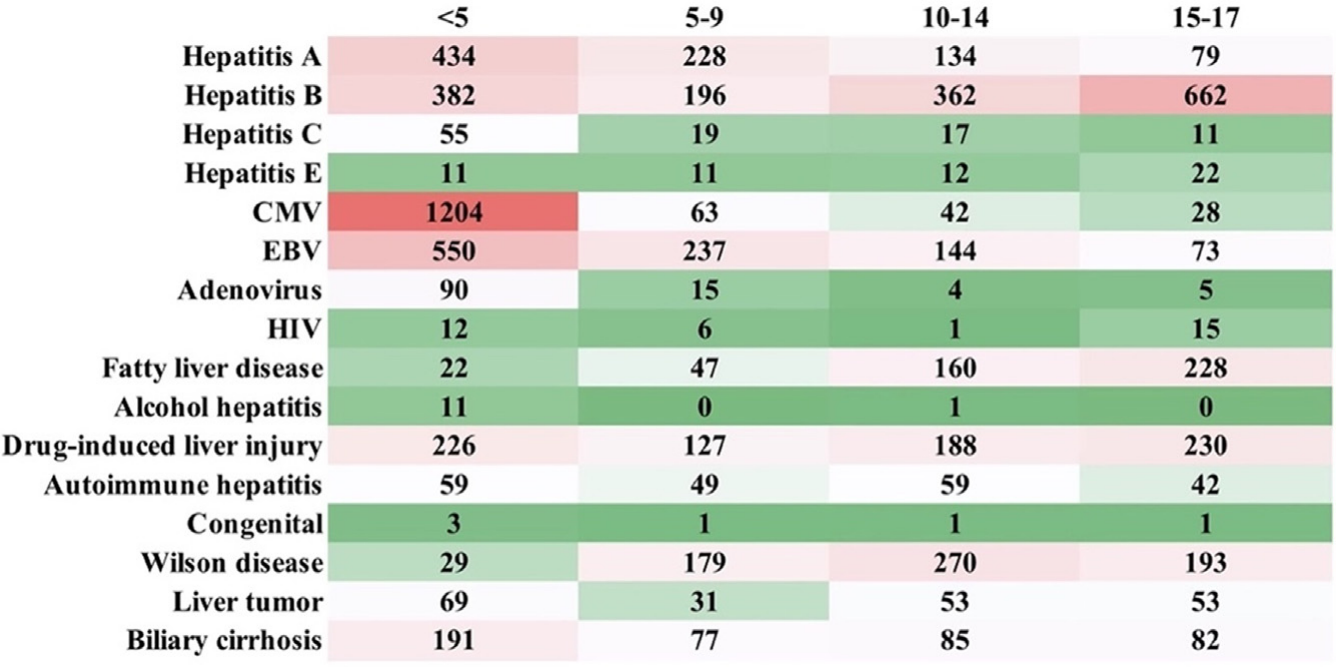
Causes of severe hepatitis among hospitalized children

Among children hospitalized for severe hepatitis (Figure 3; Table S4), 14.9% (*n* = 5090) had viral hepatitis. Hepatitis B was one of the most common causes (4.7%) and increased with age: children aged <5, 5–9, 10–14, and 15–17 years accounted for 1.9%, 3.9%, 7.5%, and 13.8%, respectively. The second most common cause was CMV infection (3.9%), which occurred mainly among children aged <5 years (6.1%). The other common causes of hospitalization for severe hepatitis among children were EBV infection (2.9%), hepatitis A (2.5%), drug-induced liver injury (2.2%), and Wilson disease (2.0%). Only 114 (0.3%) children had adenovirus infection. None of the children had a history of COVID-19 infection, or COVID-19 vaccine related hepatitis.

The common etiology of severe hepatitis varied by age. Among children aged <5 years, the most common causes for hospitalization for severe hepatitis were CMV infection (6.1%), EBV infection (2.9%), and hepatitis A (2.2%). Among those aged 5–9 years, EBV infection (4.8%), hepatitis A (4.6%), and Wilson disease (3.6%) were most common. Hepatitis B (7.5%), Wilson disease (5.6%), and drug-induced liver injury (3.9%) were most commonly found in those aged 10–14. Hepatitis B (13.8%), drug-induced liver injury (4.8%), fatty liver disease (4.7%), and Wilson disease (4.8%) were the common causes among children aged 15–17 years.

Most children hospitalized for severe hepatitis were diagnosed with indeterminate causes (79.0%). Children aged <5 years accounted for the majority (83.6%) of children hospitalized for indeterminate causes of hepatitis, and those aged 5–9 years, 10–14 years, and 15–17 years accounted for 77.3%, 72.4%, and 68.1%, respectively.

### Clinical characteristics of children hospitalized for severe hepatitis

The most common complications were respiratory failure (9.8%), hepatic encephalopathy (9.3%), coagulopathy (7.2%), and ascites (6.3%) (Table 2). The proportion of patients admitted to the ICU was 5.2%, and the median length of ICU stay was 4 days. Hemodialysis was given to 5.7% of patients, and mechanical ventilation was provided to 2.8% of patients. Furthermore, the proportions of patients with liver biopsy, liver transplantation, and artificial liver were 1.8% (*n* = 604), 1.3% (*n* = 452), and 0.7% (*n* = 245), respectively.

**Table 2:** Clinical characteristics of children hospitalized for severe hepatitis

	Total (*N* = 34410)	<5 years (*N* = 19816)	5-9 years (*N* = 4986)	10-14 years (*N* = 4803)	15-17 years (*N* = 4805)	P
Hepatic encephalopathy	3198(9.3)	1685(8.5)	569(11.4)	527(11.0)	417(8.7)	<0.001
Gastrointestinal bleeding	1946(5.7)	983(5.0)	347(7.0)	314(6.5)	302(6.3)	<0.001
Ascites	2169(6.3)	932(4.7)	373(7.5)	435(9.1)	429(8.9)	<0.001
Coagulopathy	2480(7.2)	1287(6.5)	435(8.7)	426(8.9)	332(6.9)	<0.001
Liver failure	29152(84.7)	17283(87.2)	4166(83.6)	3890(81.0)	3813(79.4)	<0.001
Acute kidney failure	1662(4.8)	609(3.1)	217(4.4)	363(7.6)	473(9.8)	<0.001
Acute respiratory failure	3364(9.8)	2188(11.0)	388(7.8)	387(8.1)	401(8.3)	<0.001
Brain death	21(0.1)	7(0)	9(0.2)	3(0.1)	2(0)	0.007
ICU stay	1774(5.2)	943(4.8)	316(6.3)	302(6.3)	213(4.4)	<0.001
Pediatric ICU stay	1028(3.0)	652(3.3)	202(4.1)	160(3.3)	14(0.3)	<0.001
Length of ICU stay (d)	4(0, 9)	3(0,10)	5(2, 10)	3(1,8)	3(1,7)	0.02
Mechanical ventilation	979(2.8)	551(2.8)	175(3.5)	150(3.1)	103(2.1)	<0.001
Mechanical ventilation duration (h)	60(14, 228)	55(13, 251)	75(18,173)	65(15,229)	68(17,171)	0.98
Haemodialysis	1966(5.7)	851(4.3)	376(7.5)	405(8.4)	334(7.0)	<0.001
Artificial liver	245 (0.7)	23(0.1)	22(0.4)	72(1.5)	128(2.7)	<0.001
Liver biopsy	604(1.8)	289(1.5)	142(2.8)	115(2.4)	58(1.2)	<0.001
Liver transplantation	452 (1.3)	325(1.6)	50(1.0)	50(1.0)	27(0.6)	<0.001
Death	2085 (6.1)	1108 (5.6)	274 (5.5)	334 (7.0)	369 (7.7)	<0.001

The percentage of patients admitted to the ICU was 4.8% among children aged <5 years, 6.3% among those aged 5–9 years, 6.3% among those aged 10–14 years, and 4.4% among those aged 15–17 years. A greater percentage of older children (aged 15–17) had artificial livers (2.7%), and a greater percentage of younger children (<5 years) had liver transplantations (1.6%).

### Outcomes and associated risk factors with mortality, hospital stay, and overall cost

The in-hospital mortality among all pediatric patients was 6.1% (*n* = 2085). The in-hospital mortality rates of children (<5 years, 5–9 years, 10–14 years, and ≥15–17 years) were 5.6%, 5.5%, 7.0%, and 7.7%, respectively (*p* < 0.001). The mortality differed according to age group, with a higher rate among children aged ≥15–17 years.

We found large differences in in-hospital mortality in different provinces, with the highest mortality reaching 16.3% and the lowest being 1.6% (Figure S1). The in-hospital mortality rate of severe hepatitis among children has substantially increased annually from 4.3% to 9.4% since 2016 (Figure 2A). Similar findings were found after the COVID-19 outbreak (RR 1.60, 95% CI 1.45 to 1.75, *p* < 0.001) and after COVID-19 vaccinations began (RR 1.68, 95% CI 1.42 to 1.98, *p* < 0.001).

**Figure 4:**
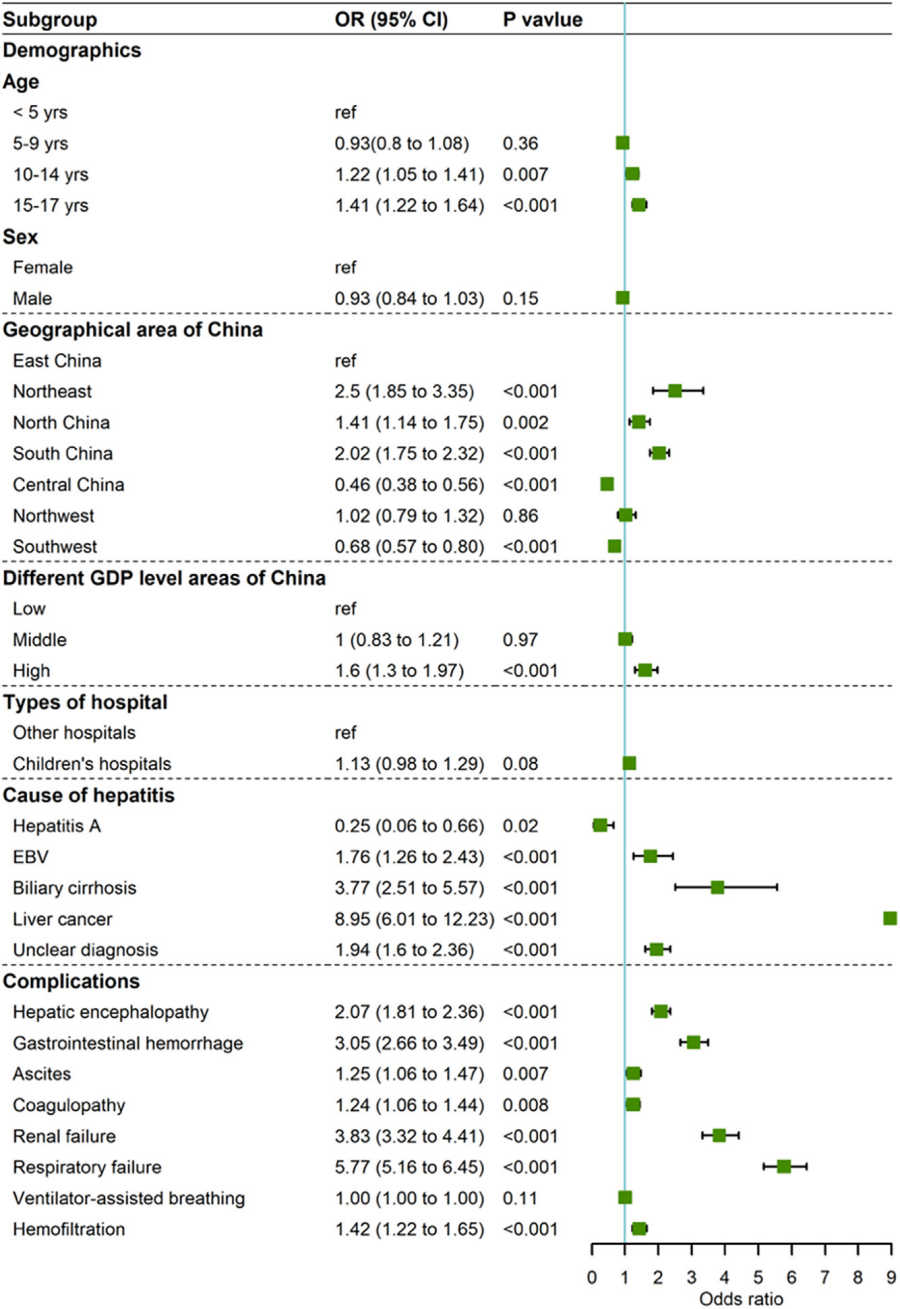
Risk factor analysis for in-hospital mortality of children hospitalized for severe hepatitis

Using the baseline data, causes and complications of children hospitalized for severe hepatitis, we performed binary logistic multivariate analysis to elucidate the risk factors for in-hospital mortality (Figure 4). Age (≥10 years), residence in Northeast, North, and South China, and areas with high GDP, EBV infection, biliary cirrhosis, liver cancer, indeterminate diagnosis and complications including hepatic encephalopathy, gastrointestinal bleeding, ascites, coagulopathy, renal failure, respiratory failure, and hemofiltration correlated with a higher risk of in-hospital mortality. Residence in Central China and Southwest China and hepatitis A were associated with a lower risk of in-hospital mortality.

**Figure 5:**
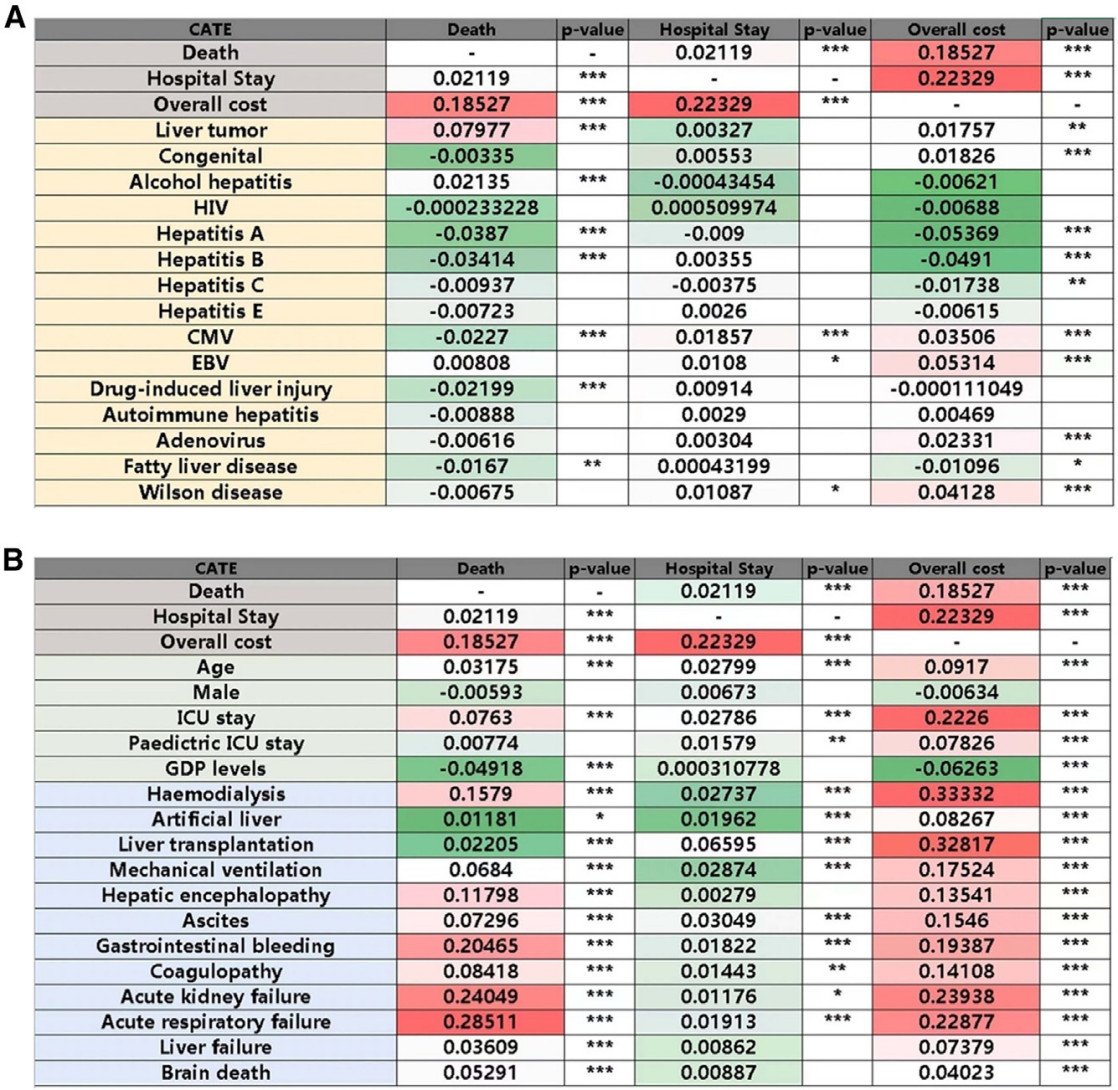
Correlation analysis of hospital stay, costs, and mortality in severe hepatitis: causes and complications (A) Correlation analysis of the length of hospital stays, overall cost and death associated with causes of severe hepatitis. (B) Correlation analysis of the length of hospital stay, overall cost and death associated with complications of severe hepatitis. ∗*p* < 0.05,∗∗*p* < 0.01,∗∗∗*p* < 0.001.

There were positive correlations between hospital stay and death (r = 0.02, *p* < 0.001), and overall cost and death (r = 0.19, *p* < 0.001). The length of hospital stay was positively correlated with age, ICU stay, GDP levels, artificial liver, liver transplantation, mechanical ventilation, ascites, gastrointestinal bleeding, coagulopathy, acute kidney failure, acute respiratory failure, CMV infection, EBV infection, and Wilson disease (r = 0.01–0.22, *p* < 0.05). Similarly, there were positive correlations of the overall cost with liver tumor, congenital severe hepatitis, CMV infection, EBV infection, Wilson disease, adenovirus, and complications (r = 0.02–0.33, *p* < 0.05) (Figure 5).

## Discussion

This study represents the first nationwide epidemiological analysis of children hospitalized with severe hepatitis in mainland China and, to our knowledge, is the largest such study globally. The data are real-world data and can well reflect the epidemiological characteristics and in-hospital mortality associated with severe hepatitis in children. Despite ongoing public health efforts, the incidence of hospitalization for severe hepatitis among children has remained consistent across the years. Viral hepatitis (notably hepatitis B, CMV infection, EBV infection, and hepatitis A), drug-related liver injury, and Wilson disease were identified as the most common recognized causes. Notably, the percentages of hospitalized children with hepatitis B increased with age: those aged <5, 5–9, 10–14, and 15–17 years accounted for 1.9%, 3.9%, 7.5%, and 13.8% of the sample, respectively. Furthermore, the majority of cases (79.0%) had indeterminate causes, particularly among children younger than 5 years, which was a significant risk factor for in-hospital mortality.

We first provided an unprecedented national perspective on the different etiological components of pediatric severe hepatitis hospitalization. For children aged <5 years, the most common recognized causes of hospitalization for severe hepatitis were viral hepatitis, including CMV infection, EBV infection, and hepatitis A. For children aged 5–9 years, EBV infection, hepatitis A and B, Wilson disease, and drug-induced liver injury emerged as key causes. With regard to children aged ≥10 years, hepatitis B, Wilson disease, drug-induced liver injury, and fatty liver disease were more prominent. These age-specific patterns offer valuable guidance for clinicians in the differential diagnosis and management of severe hepatitis in children across different age groups.

Although hepatitis B vaccination has been widely implemented in China since 2002,^5^ we found that hepatitis B remained the most common cause (4.7%) among the recognized causes of severe hepatitis in hospitalized children. Notably, the prevalence of hepatitis B among hospitalized children increased with age. National serologic surveys conducted in 2014 demonstrated that the prevalence of hepatitis B surface antigen (HBsAg)-positive individuals aged 1–4, 5–14, and 15–29 years was 0.3%, 0.9%, and 4.4%, respectively.^6^ However, we found that hepatitis B was still a common cause of hepatitis among hospitalized children (4.7%), from 1.9% among children aged <5 years to 13.8% among older children aged 15–17 years, which was higher than the prevalence reported in the general Chinese population.^6^ Older children may experience waning immunity due to a decrease in protective anti-HBs antibodies, which can leave them more vulnerable to hepatitis B infections. This is especially critical for those living in endemic areas, where exposure risks are higher.

To address this, we have emphasized the need for monitoring hepatitis B surface antigen (HBsAg) status in older children and outlined the importance of considering booster doses to maintain long-term immunity in this vulnerable group.

This elevated prevalence in our cohort may be attributed to a lack of periodic serological monitoring following primary hepatitis B vaccination and subsequent infection after the loss of protective anti-HB antibodies. To mitigate this risk, it is crucial to monitor anti-HB antibodies levels in children aged 3–7 years and provide a booster dose every 3–5 years if necessary.^7^^,^^8^ In areas with a high prevalence of hepatitis B, all children should be considered at high risk, and regular antibody monitoring with timely revaccination should be prioritized. Such public health strategies could significantly enhance the long-term effectiveness of hepatitis B vaccination in preventing infections. As economic conditions improve, regular serological monitoring and booster vaccinations become more feasible, in order to maintain immunity and prevent potential HBV infection. This proactive surveillance and vaccination strategy could be extended to other hepatitis B-endemic regions globally, contributing to the achievement of WHO’s elimination goals by 2030.^9^

Our study underscores the importance of expanded surveillance and etiological testing, a recommendation echoed by previous research with smaller or region-specific samples.^7^^,^^8^ Notably, prior studies have highlighted the decline in seroprevalence of hepatitis antibodies with age, prompting proposals for alternative vaccination strategies. By analyzing a large, nationally representative cohort, our study offers critical insights into regional variations and subpopulations that may benefit from tailored vaccination programs and earlier interventions. Emerging evidence also suggests the potential involvement of novel pathogens or environmental factors in the etiology of pediatric hepatitis, particularly in regions with unique epidemiological profiles. Future studies should explore these possibilities using advanced analytical techniques, such as multivariate models incorporating environmental data, co-infection rates, and socioeconomic factors. Such approaches could enhance our understanding of the multifactorial nature of pediatric hepatitis and inform more targeted public health strategies.

In addition to the recognized viral causes, CMV and EBV infections were also prominent in our cohort. CMV infection was particularly common among children under 5 years, consistent with its role as a leading cause of congenital infections.^10^ An increase in anti-CMV immunoglobulin G (IgG) seroprevalence among younger children has been observed in recent years.^11^ We should improve the screening for CMV infection and early intervention could improve outcomes in this population. EBV infection, on the other hand, was an independent risk factor for higher in-hospital mortality. This may be explained by the relationship of EBV infection with hemophagocytic lymphohistiocytosis^12^ and EBV-associated lymphoproliferative disorders.^13^

The high prevalence of indeterminate causes (79.0%) is particularly concerning, as it is associated with significantly higher mortality. Previous studies on pediatric acute liver failure (ALF) have reported indeterminate causes in 30–60% cases in North America^14^ and 46.8% in China.^15^ The low rate of liver biopsy (1.8%) and absence of next-generation sequencing (NGS) analyses in our study may partially explain the high proportion of indeterminate cases. Given the safety and diagnostic value of liver biopsy, especially in those with ALF,^16^^,^^17^ we recommend more routine use of biopsy and NGS to identify rare or atypical pathogens, such as herpesviruses, parainfluenza viruses, and coxsackievirus,^18^ which may be underdiagnosed.

We need to pay close attention to children with a high risk of mortality, including older children (>10 years), geographic location (Northeast, North, and South China), and specific clinical conditions (e.g., EBV infection, biliary cirrhosis, liver cancer, and indeterminate causes). Clinicians should provide close follow-up high-risk patients and consider liver transplantation when appropriate. A lower rate of children (1.3%, 452/34410) who received liver transplantation was observed, consistent with previous study in China (3.13%, 1/32).^15^ Liver transplantation can improve the prognosis of children with liver failure. A meta-analysis demonstrated that the one-year and five-year survival rates of children with liver transplantation were 86.6% and 74.0%, respectively.^19^ Given the survival benefits of liver transplantation, a more proactive approach to evaluating and referring candidates could improve outcomes.

Our data showed a decline in the number of children hospitalized for severe hepatitis after the COVID-19 outbreak. Nevertheless, in-hospital mortality has increased in recent years and was 9.4% from January through April 2022. The reason for the inconsistency was explained by the following: strained healthcare resources, reduced access to hospital care, and delayed presentations of severe cases. Specialized surveillance systems are needed to better monitor and manage pediatric severe hepatitis, particularly in the context of shifting healthcare landscapes.

### Conclusion

In conclusion, while hepatitis B is still a leading cause of hospitalization for severe hepatitis among children, more robust monitoring and booster vaccination strategies are needed to sustain long-term immunity. Enhanced etiological diagnostics, including liver biopsy and NGS, should be incorporated into clinical practice to address the high prevalence of indeterminate cases. A dedicated supervision system and predictive models for mortality could improve outcomes for high-risk children, ultimately contributing to better management and prevention strategies.

### Limitations of the study

This is the first comprehensive study among children hospitalized for severe hepatitis in mainland China, from a large-scale, nationally representative inpatient sample. We have highlighted critical patterns that can inform public health strategies and clinical practices. However, there are several limitations as following. First, the data predominantly originate from larger hospitals, which may limit the representation from rural or remote healthcare facilities. Although the study covers a large national cohort, some diagnostic procedures, such as liver biopsy and NGS, were not systematically applied. As a result, a significant proportion of cases (79%) were indeterminate cause, limiting to make definitive conclusions about specific causes of severe hepatitis. Future studies could incorporate more advanced diagnostic tools to identify underlying etiologies. Second, the HQMS database includes children admitted to hospitals, except the cases with mild hepatitis or those treated in outpatient settings. Thus, our study focuses on more severe cases, potentially limiting the generalizability of the findings to all pediatric hepatitis cases in China. Moreover, we also recognize that other factors, such as socioeconomic status and environmental exposures, may influence outcomes, were not available in our dataset. Future research could incorporate these variables to provide a more comprehensive understanding of risk factors for children with severe hepatitis.

Additionally, detailed clinical information on the treatment and prognoses is lacking. Although we included children with chronic hepatitis based on ICD diagnoses, the majority (84.7%) of our cohort experienced ALF, reflecting the clinical reality in China, where only children with severe hepatitis are typically hospitalized. This aligns with the study’s focus on severe cases. However, the lack of unique patient identification numbers in the HQMS database prevents to identify repeat hospitalization or new diagnoses. Furthermore, we could not analyze patient treatment pathways combining their residence and the hospital location, limiting to track the full medical trajectory. However, the large-scale nature of the dataset mitigates the impact of individual variations. Finally, this study lacks comprehensive data on specific viral etiologies, including VZV, HPIVs, YFV, and/or human adenoviruses, which may have led to an underestimation of children hospitalized for viral hepatitis, particularly those caused by less common pathogens.

## Resource availability

### Lead contact

For additional details and inquiries regarding data sharing, please contact the lead contact, Xiang Zhou (zx_pumc@163.com).

### Materials availability

This study did not use or generate any reagents.

### Data and code availability

• **Data availability**: The datasets analyzed in this study have been deposited in the HQMS database and are publicly accessible as of the publication date. Accession details are listed in the key resources table. Data analyzed in this study can also be made available on reasonable requests to the lead contact, Xiang Zhou.
• **Code availability**: This study did not involve or generate original code.
• **Other information**: For further inquiries, please reach out to the lead contact listed in the "resource availability" section.

## Acknowledgments

Thanks to all the investigators and subjects who participated in the China Critical Care Clinical Trials Group (CCCCTG) (Table S5). This work was supported by the CAMS Innovation Fund for Medical Sciences (CIFMS) from the 10.13039/501100005150Chinese Academy of Medical Sciences (2021-I2M-1-062), 10.13039/501100012166National Key R&D Program of China, 10.13039/501100002855Ministry of Science and Technology of the People’s Republic of China (2021YFC2500801), 10.13039/501100005089Beijing Municipal Natural Science Foundation (M21019), CAMS Endowment Fund (no. 2021-CAMS-JZ004), CMB Open Competition Program (20-381) and Chinese Medical Information and Big Data Association (CHMIA) Special Fund for Emergency Projects. The funders had no role in study design, data collection and analysis, decision to publish, or preparation of the manuscript.

## Author contributions

F.G., X.M., and Q.S.: data collection, data analysis, data interpretation, and drafting the manuscript. Y.Z.: data analysis, S.G., J.C., Y.C., and D.L.: data collection and interpretation and critically revised the manuscript for important intellectual content. T.L., F.Z., and X.Z.: study concept and design, acquisition of data, data analysis, critical review and revision of the manuscript for important intellectual content.

## Declaration of interests

The authors declare that they have no competing interests.

## STAR★Methods

### Key resources table

**Table:** 

REAGENT or RESOURCE	SOURCE	IDENTIFIER
**Deposited data**		

Hospital Quality Monitoring System (HQMS)	National Health Commission of the People’s Republic of China	https://www.hqms.org.cn/login.jsp

**Software and algorithms**		

R program	R Foundation for Statistical Computing, Vienna, Austria	https://www.R-project.org/

### Experimental model and study participant details

All data used in this study were obtained from the Hospital Quality Monitoring System (HQMS) database. In this study, we analyzed a sample of 34,410 pediatric patients (children under 18 years old) who were hospitalized for severe hepatitis. These patients were identified based on specific ICD-10 codes (B15-B19 for hepatitis and K72 for hepatic failure).

To understand clinical and demographic variations, the sample was further categorized into subgroups based on factors such as age, hepatitis type, and outcome (e.g. in-hospital mortality). Each experimental group was defined to allow for targeted comparisons—for instance, comparisons by age. The sample distribution was designed to help assess risk factors, and mortality predictors across different patient profiles within the pediatric population.

### Method details

The data were extracted from the Hospital Quality Monitoring System (HQMS), a national database containing detailed medical records of patients from secondary and tertiary hospitals across China.

#### Study population and inclusion criteria

This study included pediatric patients under the age of 18 who were hospitalized for severe hepatitis as defined by ICD-10 codes B15-19 (hepatitis) and K72(hepatic failure). Cases were selected based on the following criteria: age under 18 years and hospitalization records indicating severe hepatitis.

#### Data collection and variables

The dataset provided the following: 1) demographics (age, sex, ethnicity, and geographic region of residence), 2) clinical details (primary and secondary diagnoses, including specific hepatitis-related conditions), 3) hospitalization details (length of hospital stay, occurrence of severe complications (e.g., hepatic encephalopathy, ascites, respiratory failure)), and outcomes (in-hospital mortality). Patients were grouped by age: under 5, 5-9, 10-14, and 15-17 years. Diagnosis was based on ICD-10 coding and clinical assessments recorded by the healthcare providers in each hospital.

#### Limitations in diagnostic data

Liver biopsies and next-generation sequencing (NGS) were not systematically available in the dataset. Consequently, some cases had an indeterminate cause due to the absence of these advanced diagnostic tools, limiting the ability to confirm diagnoses through histological or genomic evidence.

### Quantification and statistical analysis

Data analysis was performed using the R software (version 4.2.1). Simple means, standard deviations, and frequencies were used to summarize demographic and clinical data. A negative binomial model was used for the incidence of hospitalization for severe hepatitis with patient numbers by province as the dependent variable and year as the independent variable. Multivariate logistic regression was used for risk factors for in-hospital mortality. The dichotomous outcome was survival versus death, and stepwise regression was applied to identify relevant risk factors using the Akaike Information Criterion (AIC). Two-tailed p-values less than 0.05 were considered statistically significant.

### Additional resources

This study is a retrospective analysis and does not involve a clinical trial; therefore, there is no clinical registry number to report.
