# Supplementary material for: Epidemiological insights into severe hepatitis in pediatric inpatients
Source: iScience. 2024 Nov 19;27(12):111420. doi: 10.1016/j.isci.2024.111420 (PMC11699462; doi:10.1016/j.isci.2024.111420)
Supplement: Document S1. Figure S1 and Tables S1–S5 [file mmc1.pdf]

iScience, Volume *27*

Supplemental information

Epidemiological insights into severe hepatitis in pediatric inpatients

Fuping Guo, Xudong Ma, Qijun Shan, Yuelun Zhang, Sifa Gao, Jieqin Chen, Yujie Chen, Dawei Liu, Taisheng Li, Feng Zhang, Xiang Zhou, and on behalf of the China Critical Care Clinical Trials Group (CCCCTG) and China National Critical Care Quality Control Center Group

Table S1. The study group was defined by the following International Classification of Diseases, Tenth Revision (ICD-10) diagnosis codes

**ICD-10**	**Diagnosis**
B15	Acute hepatitis A
B15.0	Hepatitis A with hepatic coma
B15.9	Hepatitis A without hepatic coma
B16	Acute hepatitis B
B16.0	Acute hepatitis B with delta-agent (coinfection) with hepatic coma
B16.1	Acute hepatitis B with delta-agent (coinfection) without hepatic coma
B16.2	Acute hepatitis B without delta-agent with hepatic coma
B16.9	Acute hepatitis B without delta-agent and without hepatic coma
B17	Other acute viral hepatitis
B17.0	Acute delta-(super)infection in chronic hepatitis B
B17.1	Acute hepatitis C
B17.2	Acute hepatitis E
B17.8	Other specified acute viral hepatitis
B17.9	Acute viral hepatitis, unspecified
B18	Chronic viral hepatitis
B18.0	Chronic viral hepatitis B with delta-agent
B18.1	Chronic viral hepatitis B without delta-agent
B18.2	Chronic viral hepatitis C
B18.8	Other chronic viral hepatitis
B18.9	Chronic viral hepatitis, unspecified
B19	Unspecified viral hepatitis
B19.0	Unspecified viral hepatitis with hepatic coma
B19.9	Unspecified viral hepatitis without hepatic coma
K72.0	Acute and subacute hepatic failure
K72.1	Chronic hepatic failure
K72.9	Hepatic failure, unspecified

Table S2. Causes and complications of children hospitalized for severe hepatitis by the following International Classification of Diseases, Tenth Revision (ICD-10) diagnosis codes.

ICD-10	Diagnosis
B25, B27.1, P35.1, P35.1	CMV infection
B27.0	EBV infection
B34.0	Adenovirus infection
B20-24	HIV infection
K76.0	Fatty liver disease
K70	Alcohol hepatitis
K71	Drug-induced liver injury
K75.4	Autoimmune hepatitis
K76.8	Congenital
E83.0	Wilson disease
C22, D13.4	Liver tumor
K74.5	Biliary cirrhosis
K72.903	Hepatic encephalopathy
K92.2	Gastrointestinal bleeding
R18	Ascites
D68.9	Coagulopathy
K72, K70.4, K71.1	Liver failure
N17	Acute kidney failure
J96, P28.5	Acute respiratory failure
G93.8	Brain death
Z99.1	Mechanical ventilation
Z49.1	Haemodialysis
Z94.4	Liver transplantation

Table S3. Regional division of China

Geographical area of China	
East China	Shanghai City, Jiangsu, Zhejiang, Anhui, Fujian, Jiangxi, and Shandong provinces, as well as Taiwan (not analyzed in this survey)
South China	Guangdong Province, Guangxi Zhuang Autonomous Region (Guangxi), Hainan Province, as well as Hong Kong and Macao (not analyzed in the survey)
North China	Beijing, Tianjin, Hebei Province, Shanxi Province, and Inner Mongolia Autonomous Region (Inner Mongolia)
Central China	Henan, Hunan, and Hubei provinces
Southwest	Sichuan, Guizhou, and Yunnan provinces, Chongqing City, and the Tibet Autonomous Region (Tibet)
Northwest	Shaanxi, Gansu, and Qinghai provinces, Ningxia Hui Autonomous Region (Ningxia), and the Xinjiang Uygur Autonomous Region (Xinjiang)
Northeast	Heilongjiang, Jilin, and Liaoning provinces
Economic zones of China	
Southeast coastal area	Beijing, Tianjin, Hebei, Liaoning, Shanghai, Jiangsu, Zhejiang, Fujian, Shandong, Guangdong, Guangxi, Hainan, and Chongqing
Central inland area	Shanxi, Inner Mongolia, Jilin, Heilongjiang, Anhui, Jiangxi, Henan, Hubei, and Hunan
Western remote area	Sichuan, Guizhou, Yunnan, Tibet, Shaanxi, Gansu, Qinghai, Ningxia, and Xinjiang
Different GDP level areas of China	
High	Shanghai, Beijing, Jiangsu, Fujian, Zhejiang, Tianjin, Guangdong, Chongqing, Hubei, Henan, and Sichuan Provinces
Middle	Hebei, Hunan, Shandong, Anhui, Liaoning, Shaanxi, Jiangxi, Guangxi, and Yunnan provinces
Low	Inner Mongolia, Shanxi, Heilongjiang, Jilin, Guizhou, Xinjiang, Gansu, Hainan, Ningxia, Qinghai and Tibet Provinces

All the data are from China's new economic white paper 2021

Table S4. Causes of children hospitalized for severe hepatitis.

	Total (N=34410)	<5 (N=19816)	5-9 (N=4986)	10-14 (N=4803)	15-17 (N=4805)	P
Hepatitis A	875(2.5)	434(2.2)	228(4.6)	134(2.8)	79(1.6)	<0.001
Hepatitis B	1602(4.7)	382(1.9)	196(3.9)	362(7.5)	662(13.8)	<0.001
Hepatitis C	102(0.3)	55(0.3)	19(0.4)	17(1.4)	11(0.2)	0.44
Hepatitis E	56(0.2)	11(0.1)	11(0.2)	12(0.2)	22(0.5)	<0.001
CMV infection	1337(3.9)	1204(6.1)	63(1.3)	42(0.9)	28(0.6)	<0.001
EBV infection	1004(2.9)	550(2.8)	237(4.8)	144(3.0)	73 (1.5)	<0.001
Adenovirus infection	114(0.3)	90 (0.5)	15(0.3)	4(0.1)	5(0.1)	<0.001
HIV infection	34(0.1)	12(0.1)	6 (0.1)	1 (0)	15(0.3)	<0.001
Fatty liver disease	457 (1.3)	22(0.1)	47(0.9)	160(3.3)	228(4.7)	<0.001
Alcohol hepatitis	12 (0.03)	11(0.03)	0	1(0)	0	0.13
Drug-induced liver injury	771(2.2)	226 (1.1)	127(2.5)	188(3.9)	230(4.8)	<0.001
Autoimmune hepatitis	209 (0.6)	59 (0.3)	49 (1.0)	59 (1.2)	42 (0.9)	<0.001
Congenital	6 (0.02)	3 (0.01)	1 (0)	1 (0)	1 (0)	0.87
Wilson disease	671 (2.0)	29 (0.1)	179(3.6)	270(5.6)	193 (4.0)	<0.001
Liver tumor	206(0.6)	69(0.3)	31(0.6)	53(1.1)	53(1.1)	<0.001
Biliary cirrhosis	435 (1.3)	191(1.0)	77(1.5)	85(1.8)	82(1.7)	<0.001
Unclear diagnosis	27175(79.0)	16572(83.6)	3855(77.3)	3476(72.4)	3272(68.1)	<0.001

Table S5. China Critical Care Clinical Trials Group (CCCCTG) Members

No.	Affiliations	Department	Contact
1	The First Affiliated Hospital of Harbin Medical University	Department of Critical Care Medicine	Mingyan Zhao, Songlin Yang
2	The First Affiliated Hospital of China Medical University	Department of Critical Care Medicine	Xiaochun Ma, Yini Sun
3	The Second Hospital of Jilin University	Department of Critical Care Medicine	Yongjie Yin, Dexin Liu
4	Hebei Medical University Fourth Hospital	Department of Critical Care Medicine	Zhenjie Hu, Yan Huo
5	Fuxing Hospital, Capital Medical University	Department of Critical Care Medicine	Li Jiang, Qi Zhang
6	Peking University People's Hospital	Department of Critical Care Medicine	Youzhong An, Huiying Zhao
7	Beijing Tongren Hospital, Capital Medical University	Department of Critical Care Medicine	Yuan Xu, Wei He
8	Qilu Hospital of Shandong University	Department of Critical Care Medicine	Dawei Wu, Chen Li
9	Ruijin Hospital, Shanghai Jiao Tong University	Emergency Intensive Care Unit	Enqiang Mao, Cheng Zhu
10	Guangdong Geriatric Institute, Guangdong General Hospital	Department of Critical Care Medicine	Tiehe Qin, Shouhong Wang
11	The First Affiliated Hospital, Zhengzhou University	Surgical Intensive Care Unit	Rongqing Sun, Yuexia Li
12	Xiangya Hospital, Central South University	Department of Critical Care Medicine	Yuhang Ai, Yin Huang
13	The First Affiliated Hospital of Fujian Medical University	Department of Critical Care Medicine	Jiandong Lin, Xiongjian Xiao
14	Hainan Provincial People's Hospital	Department of Critical Care Medicine	Zhenyang He, Rui Li
15	West China Hospital, Sichuan University	Department of Critical Care Medicine	Yan Kang, Xuelian Liao
16	Tongji Hospital of Tongji Medical College, Huazhong University of Science & Technology	Department of Critical Care Medicine	Shusheng Li, Xiao Ran
17	First Affiliated Hospital, Xinjiang Medical University	Department of Critical Care Medicine	Xiangyou Yu, Yi Wang
18	The Affiliated Hospital of Inner Mongolia Medical University	Department of Critical Care Medicine	Lihua Zhou, Lipeng Zhang
19	Xijing Hospital, Fourth Military Medical University	Surgical Intensive Care Unit	Xijing Zhang, Binxiao Su

20	The First Affiliated Hospital of Chongqing Medical University	Department of Emergency and Intensive Care Medicine	Fachun Zhou, Fang Xu
21	Zhejiang Provincial People's Hospital	Department of Critical Care Medicine	Renhua Su, Qian Li
22	The First Affiliated Hospital of Kunming Medical University	Department of Emergency Medicine and Emergency/Medical ICU	Chuanyun Qian, Wei Zhang
23	Peking Union Medical College Hospital	Medical Intensive Care Unit	Bin Du, Li Weng, Xiaoyun Hu
24	General Hospital of Ningxia Medical University	Department of Critical Care Medicine	Xiangyuan Cao, Xigang Ma
25	China-Japan Friendship Hospital	Medical Intensive Care Unit, Department of Pulmonology	Qingyuan Zhan, Xu Huang
26	Qinghai University Affiliated Hospital	Emergency Intensive Care Unit	Ming Hou

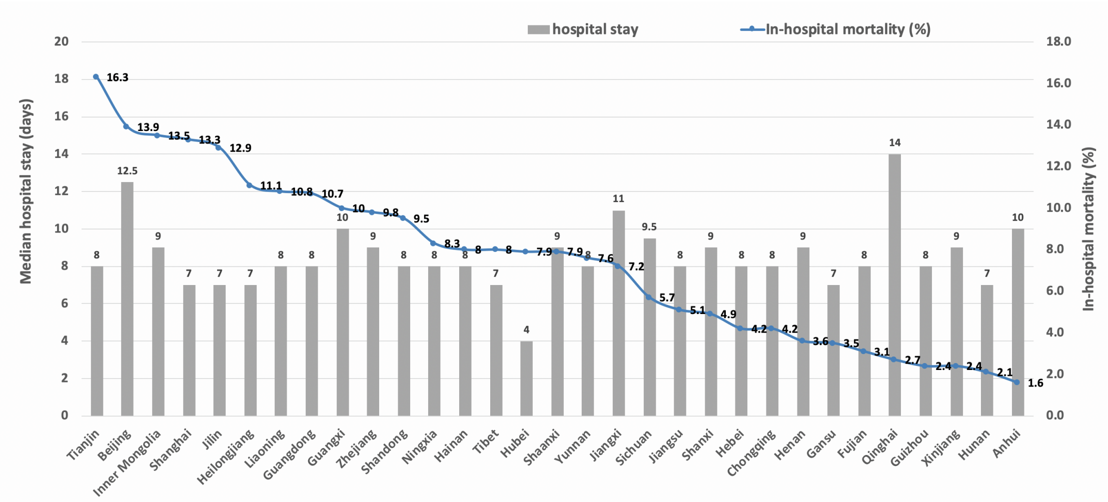

Figure S1. Hospital stay and in-hospital mortality rates of children hospitalized for severe hepatitis in different provinces.
